# Molecular Changes in Pre-Metastatic Lymph Nodes of Esophageal Cancer Patients

**DOI:** 10.1371/journal.pone.0102552

**Published:** 2014-07-21

**Authors:** Benjamin Otto, Alexandra M. Koenig, Genrich V. Tolstonog, Anke Jeschke, Kristin Klaetschke, Yogesh K. Vashist, Daniel Wicklein, Christoph Wagener, Jakob R. Izbicki, Thomas Streichert

**Affiliations:** 1 Department of Internal Medicine, Center for Internal Medicine, University Medical Center Hamburg-Eppendorf, Hamburg, Germany; 2 Department of Clinical Chemistry, Center for Diagnostic, University Medical Center Hamburg-Eppendorf, Hamburg, Germany; 3 Department of General, Visceral and Thoracic Surgery, Center for Surgical Sciences, University Medical Center Hamburg-Eppendorf, Hamburg, Germany; 4 Department of Otolaryngology – Head and Neck Surgery, CHUV, University of Lausanne, Lausanne, Switzerland; 5 Department of Osteology and Biomechanics, Center for Experimental Medicine, University Medical Center Hamburg-Eppendorf, Hamburg, Germany; 6 Department of Anatomy and Experimental Morphology, Center for Experimental Medicine, University Medical Center Hamburg-Eppendorf, Hamburg, Germany; The University of Hong Kong, China

## Abstract

Lymph node metastasis indicates poor prognosis in esophageal cancer. To understand the underlying mechanisms, most studies so far focused on investigating the tumors themselves and/or invaded lymph nodes. However they neglected the potential events within the metastatic niche, which precede invasion. Here we report the first description of these regulations in patients on transcription level. We determined transcriptomic profiles of still metastasis-free regional lymph nodes for two patient groups: patients classified as pN1 (n = 9, metastatic nodes exist) or pN0 (n = 5, no metastatic nodes exist). All investigated lymph nodes, also those from pN1 patients, were still metastasis-free. The results show that regional lymph nodes of pN1 patients differ decisively from those of pN0 patients – even before metastasis has taken place. In the pN0 group distinct immune response patterns were observed. In contrast, lymph nodes of the pN1 group exhibited a clear profile of reduced immune response and reduced proliferation, but increased apoptosis, enhanced hypoplasia and morphological conversion processes. DKK1 was the most significant gene associated with the molecular mechanisms taking place in lymph nodes of patients suffering from metastasis (pN1). We assume that the two molecular profiles observed constitute different stages of a progressive disease. Finally we suggest that DKK1 might play an important role within the mechanisms leading to lymph node metastasis.

## Introduction

In esophageal cancer (ESC), lymph node metastasis is associated with poor prognosis. During the migration of tumor cells through lymph vessels and during their homing in lymph nodes, these tumor cells get into close contact to endothelial cells and lymph node sinus. Tumor expansion currently forms the main basis for therapy selection. But despite the high prognostic relevance of lymphogenic metastases, the molecular mechanisms underlying this metastatic pathway are still mostly unknown.

A number of studies investigated the molecular profiles of the tumors[1]–[4] and/or the metastatic lymph nodes.[5] Further studies investigated lymphogenic metastasis in correlation with lymphatic angiogenesis[6]–[9] and chemokine associated[10]–[12] migration. It was assumed that directed migration of esophageal carcinoma cells might be guided by locally expressed chemokines.[12] And based on these findings it would be imaginable that the homing process into the lymph nodes might be assisted by lectin-glycan-interactions between the tumor cells and lymph endothelial cells. Hirakawa et al.[13] demonstrated that in mice even before metastasizing, primary tumors (skin cancer) might stimulate sentinel lymph node lymphangiogenesis. They suggested that primary tumors could be capable of preparing their future metastatic niche by producing lymphangiogenic factors that might mediate their efficient transport to the sentinel lymph nodes.

Yet, to the authors' best knowledge there is no published work specifically examining the early molecular mechanisms in this metastatic niche in patients – mechanisms within the non-affected lymph nodes, which could propagate or suppress early metastasis in patients suffering from ESC.

The aim of this study therefore was to specifically investigate these early alterations in lymph nodes prior to histopathologically detectable metastasis. The work has been driven by the hypothesis that before tumor cells metastasize to regional lymph nodes, important changes must have already taken place propagating or suppressing the homing process. To get insight into this process, we collected tumor regional and distant lymph nodes from patients suffering from ESC staged as pN0 (no metastatic nodes exist) or pN1 (metastatic nodes exist). All lymph nodes included in the analysis were metastasis-free – irrespective of the patient staging. We performed microarray analyses and investigated the mechanisms linked to the genes differentially expressed between regional and distant lymph nodes of the same patient.

Key to our work here are two features. First, all analyzed lymph nodes without exception, even those of the pN1 patients, were still free of metastasis. Second, we used samples of patients who did not undergo any neoadjuvant radio-chemotherapy and established further procedures that ensured very high sample quality.

## Materials and Methods

### General analysis workflow

The included patients suffered from esophageal cancer and underwent a radical thoraco-abdominal en-bloc esophagectomy including lymph node dissection of the upper and lower mediastinum (R0 resection) without neoadjuvant radio-chemotherapy. We included only lymph nodes from patients staged as pN0 (regional lymph nodes free of metastasis) or pN1 (regional lymph nodes metastasis present) and verified by histopathology that the lymph nodes selected for our analysis were all tumor cell free. We also confirmed by immunohistology that these lymph nodes were free of micro-metastasis. We isolated from each lymph node total RNA for microarray expression analysis. A paired T-test was used for the identification of genes that were differentially expressed between regional and distant lymph nodes and a functional enrichment and network analysis was performed. Subsequently major candidates were validated via real time PCR and DKK1 was validated in addition via immunohistological staining.

### Experiment design

**Figure 1:**
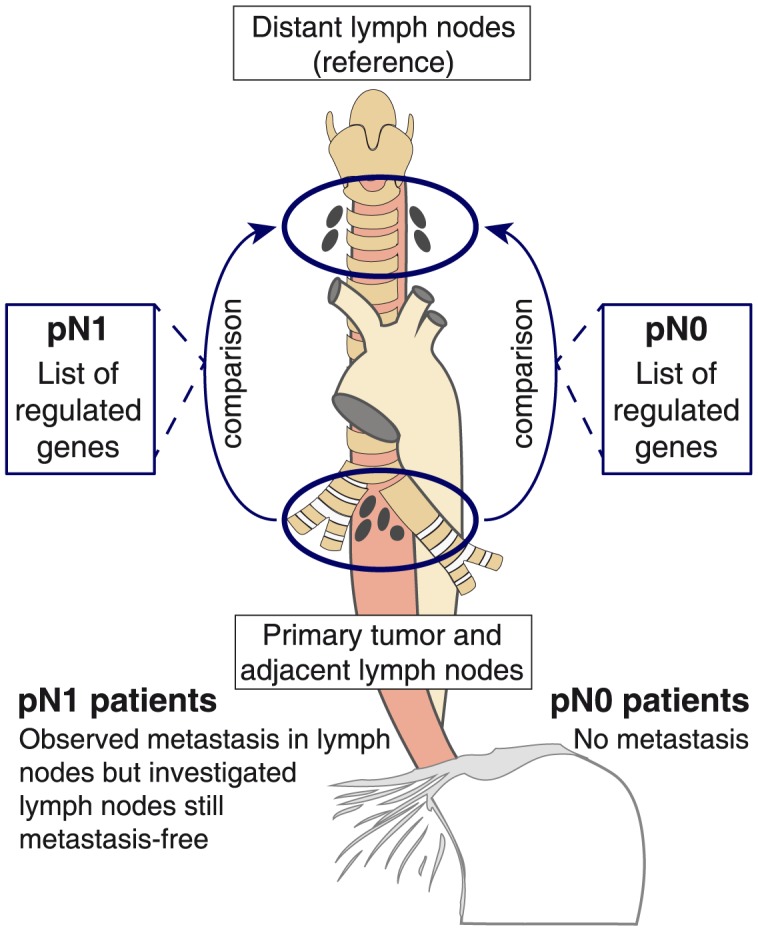
Experimental design. Lymph nodes of two different patient groups suffering (pN1) or not suffering (pN0) from lymph node metastasis were investigated. From each patient regional lymph nodes (adjacent to the tumor) were compared to distant lymph nodes (reference as baseline). Key to the experiment is that *all* investigated lymph nodes, including those of pN1 patients, were made sure to be still free of metastasis as well as micro-metastases.

The aim of the work presented here was to identify potential factors facilitating and driving metastasis in lymph nodes of patients suffering from esophageal carcinoma. To enable such understanding the experiment design (Fig. 1) incorporated two variables of interest.

First, samples were classified according to the principal status of the patients exhibiting (pN1 group) or non-exhibiting (pN0 group) metastasis in regional lymph nodes. The comparison of pN1 and pN0 patients should highlight the general differences in regulations that might lead to or prevent from metastasis.

Second, two lymph node samples were collected from each patient, one node located close to the tumor (regional) and the second node distant to the tumor. While the distant nodes served as reference sample the regional nodes were investigated for transcriptional regulations. The pairwise comparison of the tumor distant samples with the patient specific regional nodes should reveal the early regulations potentially resulting from the location close to the tumor. In this publication the term “up-regulation” is used for those genes and functions that exhibit a higher expression in the regional lymph nodes in comparison to the distant lymph nodes. Conversely, those genes and functions that are decreased in the regional lymph nodes are termed “down-regulated”.

It is important to note that all nodes investigated were made sure to be still metastasis free, independent of the patients' pN1/pN0 classification and of the location relative to the tumor. This should allow the identification of early changes occurring *prior* to metastasis homing.

### Ethics committee approval & patients consent

This study was performed in accordance with the applicable regulations and was approved by the Hamburg Ethics Committee, Germany, under vote number PV3548. Patients written consent to participate in the study was collected in accordance with the applicable regulations.

### Sample material

Overall samples from 22 patients with esophagus carcinoma were analyzed of which 14 sample pairs exhibited an adequate sample quality. For each patient sample pairs of tumor close and tumor distant lymph nodes were collected to reduce inter-individual differences that might occur due to diverging genetic background. Lymph nodes were immediately dissected upon extraction in the operation room. The segments that were dedicated for the microarray expression analysis were directly transported in liquid nitrogen to the laboratory for RNA extraction. The other part of the lymph nodes was embedded in paraffin for classification and diagnosis.

### Operation procedure

After histological diagnosis of an esophageal carcinoma, patients with early stage lesions (pT1a) were referred to surgery either if the endoscopic resection was incomplete or the tumor was staged as pT1b. Patients were directly referred to surgery if the preoperative staging was suspicious for either muscular wall involvement (cT2) or lymph node involvement (cN1). No patient was submitted to neoadjuvant therapy.

All patients underwent median T-shaped laparatomy and right-sided thoracotomy. For patients with collar anastomosis an additional left-sided cervicotomy for collar anastomosis was performed. Every patient received a radical en bloc esophagectomy with resection of the esophagus, thoracic duct, azygos vein, ipsilateral pleura, and all periesophageal tissue in the posterior mediastinum. Reconstruction was performed by gastric tube or colon interposition usually with a high intrathoracic or cervical anastomosis.

Additionally, a standardized two-field lymphadenectomy was performed. All lymph nodes were systematically sampled from eight locations: regional mediastinal, both in the vicinity of the tumor and distant from it; infraclavicular; diaphragmatic (lower mediastinal); perigastric lymph nodes (including left and right pericardial); common hepatic artery lymph nodes; and lymph nodes at the celiac trunk. All nodes were mapped by the surgeon according to the scheme of the American Thoracic Society as modified by Casson et al.[14] All resected specimens were assessed by a senior pathologist.

Postoperative follow-up was carried out as part of the usual aftercare. Patients were seen at the outpatient clinic at 3- to 4-month intervals for the first 2 years and at 6-month intervals thereafter. Follow-up evaluation included physical examination, plain chest radiography, abdominal ultrasonography, endoscopy, endosonography, CT of the chest and abdomen, with PET-CT after January 2006 in selected cases, CEA and CA 19-9 tumor marker studies, and bone scans. Whenever relapse was suspected, additional radiologic, endoscopic, or histologic confirmation was sought.

Recurrence was diagnosed if proven by biopsy or by unequivocal evidence of tumor masses (newly appearing metastases or local recurrence) with a tendency to grow during further follow-up and/or follow-up until death. Recurrent disease was defined as either locoregional (occurring in the upper abdomen or mediastium) or as distant metastasis.

### Micrometastasis staining

Hematoxylin and eosin staining was used for tumor cell detection in a first diagnostic step. Histologically “tumor-free” lymph nodes were then screened immunohistochemically using anti–cytokeratin antibody AE1/AE3 (Dako, Glostrup, Denmark) diluted 1∶50 (v/v) by the biotin-streptavidin method as described previously.[15] AE1/AE3 is a cocktail of two antibodies that recognizes basic and acidic CK's on the surface and in the cytoplasm of all epithelial cells (except parietal cells, hepatocytes, and the superficial layers of squamous epithelium). The antibodies do not react with mesenchymal tissue, including lymphoid tissue[16].

Formalin fixed and paraffin embedded sections of 5 to 6 µm thickness were cut at three different levels in each node and deparaffinized according to standard histological techniques and transferred onto glass slides treated with 3-triethoxysilylpropylamin (Merck, Darmstadt, Germany). One section of the sample obtained at each level was stained using alkaline phosphatase–anti-alkaline phosphatase technique combined with the new fuchsine stain (Sena, Heidelberg, Germany) for the visualization reaction[17].

Sections of normal colon mucosa served as positive staining controls and isotype-matched, irrelevant murine monoclonal antibodies served as negative controls (purified immunoglobulin mouse myeloma protein for IgG1; Sigma, Deisenhofen, Germany).

The slides were evaluated in a blinded procedure by two independent investigators. In case of incongruent findings a third investigator was consulted and a consensual decision was made. Minimal tumor cell involvement in a lymph node that was considered to be tumor-free by conventional histological staining was defined as the presence of one to ten positive cells in the body of the node.

### Sample preparation and purification

Isolation of total RNA from liquid nitrogen frozen lymph node samples was performed with the Trizol from Invitrogen according to the manufacturers protocol (Rev. date 12. June 2007) and was purified afterwards with the Qiagen RNeasy-Mini-Kit. Concentrations were determined with a PeqLab NanoDrop photometer and RNA quality by capillary electrophoresis using an Agilent Bioanalyzer 2100 System. Out of the 22 sample pairs 9 pairs of patients with observed far metastasis and 5 sample pairs of patients with no far metastasis observed showed an RNA quality sufficient for the following analysis.

### Microarray analysis

Microarray experiments were performed using Affymetrix human whole genome GeneChips U133 Plus 2.0 (Affymetrix, Santa Clara, USA) according the manufacturers protocol. To prepare the RNA for the expression arrays cDNA-synthesis with preceding photometric determination of the RNA concentration was performed. The following steps were performed using 250ng of total RNA for the first strand synthesis. Sample preparation incorporated steps for cDNA synthesis, purification, synthesis of the biotin-labled cRNA, in vitro transcription, fragmentation, hybridization as well as washing and staining. The preparation was performed according to the One Cycle Protocol using the GeneChip 3' IVT Express Kit from Affymetrix. For Hybridization 12.5 µg fragmented cRNA were used. The arrays were incubated for 16h in the Affymetrix Hybidization Oven 640 at 45°C. Washing and staining steps were performed semi-automatic in the Affymetrix Fluidics Station 450, the single steps following the Affymetrix One Cycle Protocol for standard arrays. After washing and staining the arrays were scanned using the Affymetrix GeneChip Scanner 3000 7G and the Command Console software at a wavelength of 570 nm and a pixel size of 1.56 µm.

### Statistical analysis

Lymph nodes from both patient groups (pN0 and pN1) were analyzed independently. As the focus of this work were early changes preceding metastasis, only metastasis free lymph nodes were analyzed. The tumor distant lymph node samples were used as reference for the paired regional lymph nodes.

Background correction and normalization were performed using RMA procedure and quantile normalization. A paired T-test within the two groups (pN0, pN1) was performed and a bootstrapping procedure was performed to adjust for false positive regulations. As cutoff values a corrected p-value of 0.05, a raw p-value of 0.01 and a signal-log-ratio (SLR) of ±0.7 were used to determine the top regulated genes.

Following differential gene expression analysis we performed gene set enrichment and network analysis with Ingenuity Pathway Analysis software (Ingenuity Systems, version 162830). For the analysis the corrected p-value cutoff was set to a less stringent value of ±0.1 to allow for the incorporation of more subtle changes.

### Validation via RT-PCR

Twelve of the determined candidate genes were validated via RT-PCR using three technical replicates per sample. The RT-PCR was performed with 96-well plates on the StepOne Plus real Time PCR System from Applied Biosystems. For each sample 2 µl with a concentration of 5 ng/µl were used. To account for fluctuations in sample concentration Beta-2-microglobulin (B2M) was taken as housekeeping reference. For determination of the regulations on expression level the mean value of the three technical replicates' delta-Ct-values was calculated. Significance of the changes was determined using a paired t-test between tumor close and tumor distant mean delta-Ct-values within each group (pN0, pN1).

### DKK1 Immunohistochemistry

Immunohistochemistry was performed on paraffin sections of the investigated lymph nodes using polyclonal antibody against DKK1 (1∶100, abcam, #ab61034). For immunohistochemical detection sections were deparaffinized, rehydrated, and pre-treated with 0.1% pronase, for 10 min for enzymatic antigen unmasking. After incubation in 3% hydrogen peroxide for 15 min to block endogenous peroxidase activity and incubation with 5% BSA for 30 min to block non-specific antibody binding primary antibody was applied. Immunohistochemical staining was performed over night at 4°C. A biotinylated secondary goat anti-rabbit IgG (1∶200, Dako Cytomation) was used, followed by incubation with a streptavidin/HRP (1∶200, Dako Cytomation) as a third antibody. Peroxidase activity was detected using DAB as chromogenic substrate (Dako Cytomation). Sections were counterstained with hematoxylin, dehydrated, and mounted. Images were collected on a Zeiss Mirax MIDI slide scanner (Zeiss) and images were captured using the Pannoramic Viewer software.

## Results

### Metastasis-free regional lymph nodes differ between pN1 and pN0 patients on transcriptomic level

We asked if a) the metastatic niche in lymph nodes might be affected even before metastasis has taken place and if b) still metastasis-free lymph nodes of pN1 patients might be more on the verge of metastatic invasion than those of pN0 patients and if c) therefore those “pN1” lymph nodes might exhibit distinct transcriptional deregulations associated with the niche preparation.

We included tumor regional lymph nodes from 14 esophageal carcinoma patients (n = 9 pN1 patients; n = 5 pN0 patients) to investigate gene expression in the potential metastatic niche. In addition we collected a paired tumor distant lymph node from each patient to serve as reference for the regional nodes (Fig. 1). Afterwards they were analyzed on transcriptomic level by microarray technology.

Key to the work presented here is that all nodes investigated were confirmed to be histopathologically and immunohistologically metastasis and micrometastasis-free, independent of patients' pN1/pN0 classification and of the location relative to the tumor. This should allow identification of early changes occurring *prior* to metastasis homing. Information about patients' age, gender, histology or classifications is provided in Table S1.

To identify the potential pre-invasive changes we compared the regional lymph nodes with the distant lymph nodes using a paired T-test within the two groups (pN0, pN1). The tumor distant lymph nodes served as reference for the regional nodes, such that up-regulations depict those genes with a higher expression level in the regional nodes and down-regulations respectively those genes with a lower expression level. The T-test was followed by a bootstrapping procedure to account for multiple testing. As cutoff values a corrected p-value of 0.05, a raw p-value of 0.01 and a signal-log-ratio (SLR) of ±0.7 were used to determine the top regulated genes. The most significant candidates identified are displayed in Table 1, complete statistics are provided in Table S2.

**Table 1:** Top regulated genes in pN0 and pN1 patients' regional lymph nodes.

*Dysregulations in pN1 regional lymph nodes*					
**Unique ID**	**raw p-value pN1**	**corr. p-value pN1**	**SLR pN1**	**SYMBOL**	**Entrez ID**
204602_at	0.0029	0.000	−1.0	DKK1	22943
202018_s_at	0.0095	0.014	−1.0	LTF	4057
235944_at	0.0076	0.005	−0.9	HMCN1	83872
202765_s_at	0.0090	0.006	−0.9	FBN1	2200
219682_s_at	0.0023	0.001	−0.8	TBX3	6926
227526_at	0.0043	0.007	−0.8	CDON	50937
231807_at	0.0085	0.006	−0.8	KIAA1217	56243
221024_s_at	0.0094	0.004	−0.7	SLC2A10	81031
238029_s_at	0.0078	0.010	−0.7	SLC16A14	151473
230087_at	0.0049	0.006	−0.7	PRIMA1	145270
206018_at	0.0068	0.004	1.4	FOXG1	2290
***Dysregulations in pN0 regional lymph nodes***					
**Unique ID**	**raw p-value pN0**	**corr. p-value pN0**	**SLR pN0**	**SYMBOL**	**Entrez ID**
202267_at	0.0058	0.000	−0.9	LAMC2	3918
223805_at	0.0018	0.008	−0.8	OSBPL6	114880
202341_s_at	0.0044	0.016	−0.7	TRIM2	23321
237390_at	0.0041	0.000	−0.7	NA	NA
202342_s_at	0.0053	0.008	−0.7	TRIM2	23321
205794_s_at	0.0058	0.000	−0.7	NOVA1	4857
204584_at	0.0085	0.008	−0.7	L1CAM	3897
227461_at	0.0042	0.000	−0.7	STON2	85439
236926_at	0.0085	0.032	−0.7	TBX1	6899
1552575_a_at	0.0068	0.024	−0.7	C6orf141	135398
219867_at	0.0088	0.000	−0.7	CHODL	140578
244655_at	0.0079	0.008	−0.7	LOC100132798	100132798
226770_at	0.0030	0.015	−0.7	MAGI3	260425
1553622_a_at	0.0065	0.000	−0.7	FSIP1	161835
219316_s_at	0.0029	0.000	0.7	FLVCR2	55640
210845_s_at	0.0022	0.000	0.8	PLAUR	5329
211924_s_at	0.0066	0.000	0.8	PLAUR	5329
203561_at	0.0029	0.000	0.8	FCGR2A	2212
204429_s_at	0.0062	0.000	0.9	SLC2A5	6518
205767_at	0.0061	0.000	1.2	EREG	2069
220088_at	0.0014	0.000	1.4	C5AR1	728
1555643_s_at	0.0085	0.015	1.5	LILRA5	353514

The table includes the unique ids (Affymetrix Probeset), raw p-values, corrected p-values (bootstrapping), signal-log-ratios and gene description.

Applying these thresholds we identified 173 transcripts (128 genes) significantly regulated between regional and distant lymph nodes of pN0-classified patients. Several of these genes were associated with immune response (such as CD64, CD32, *FPR1* and *FPR2*,[18] *IGHG1*, *IL1R2*, *CCL23*, *MSR1*, *C5AR1*, *LILRA5*,[19] or *LILRB2* [20]). Interestingly a couple of cancer and metastasis associated genes were deregulated as well (such as *L1CAM* (CD171),[21] *EREG*,[22], [23] *NOVA1*, *HPR*,[24] or *LAMC2* [25], [26]). Epiregulin, e.g., is an angiogenic factor that can propagate metastasis by enabling tumor cells to breach lung endothelial barriers and in turn by releasing them into the circulation system[22], [23].

In contrast we identified 134 dysregulated transcripts (109 genes including *IGF1*, *DKK1*, *PRIMA1* and *LTF*) in the regional lymph nodes of pN1-classified patients. Among those genes pro-proliferative and anti-apoptotic molecules were down-regulated – such as *IGF1* and *LTF* which act through regulation of the AKT signaling pathway[27] and ERK1/2 pathway[28] respectively. PRIMA1 modulates CXCR4 expression[29] – a molecule that already has been described to correlate strongly with increased lymphatic metastasis[11], [12], [29].

Notably *DKK1* exhibited the most significant deregulation (2-fold change; p-value of 0.0029). The gene was clearly down-regulated in the regional lymph nodes of pN1 patients. DKK1 is a WNT pathway inhibitor that has been shown to be of prognostic significance in diverse tumor entities such as breast cancer, lung cancer, myeloma but also in esophageal carcinoma.[30]–[38] In addition DKK1 was reported to be elevated in the serum of ESC patients[37].

**Figure 2:**
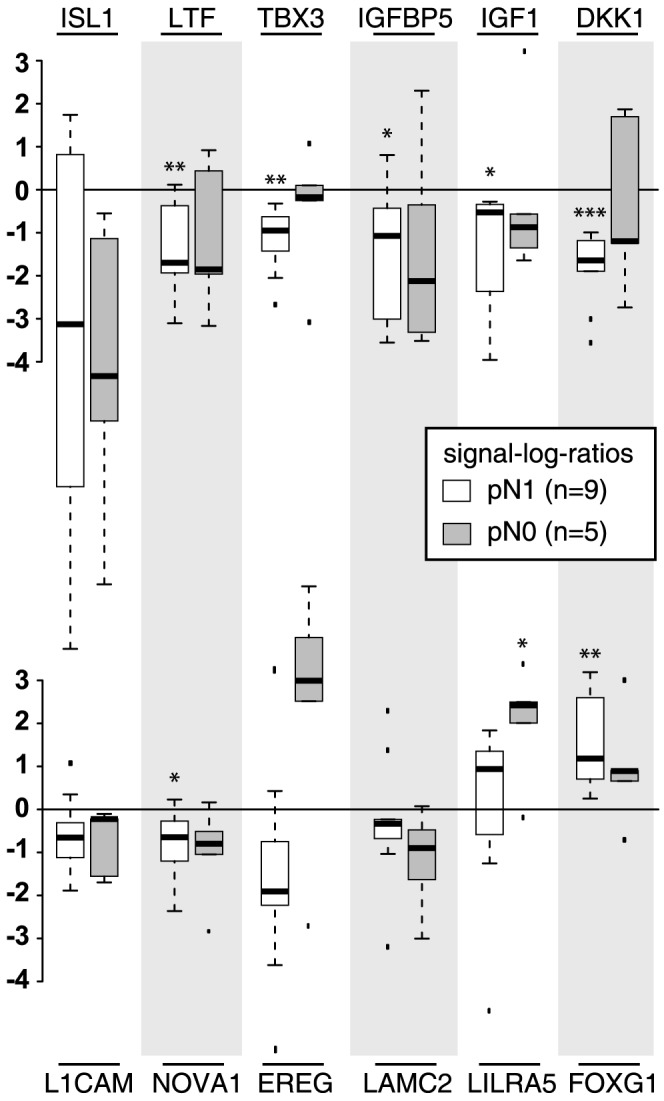
RT-PCR results for validation of microarray results. Displayed the signal-log-ratios of the regional lymph nodes in comparison to the tumor distant lymph nodes (reference) for a selected set of top regulated genes.

We validated 12 of the most significant genes via RT-PCR (Fig. 2). For five of these genes (*ISL1*, *LAMC2*, *NOVA1*, *EREG*, *L1CAM*) the regulation tendencies and strengths were confirmed though the p-values determined in the PCR analysis ranged between 0.08 and 0.15, probably due to the small sample size. Seven genes (*IGF1*, *IGFBP5*, *DKK1*, *LTF*, *TBX3*, *FOXG1*, *LILRA*) could be validated successfully showing a significant p-value below of 0.05. Highest significance (p-value of 3*10e-4) was observed again for *DKK1* (see Table S3).

By the time of follow-up analysis five pN1 patients had died as well as one pN0 patient. The latter patient had exhibited lung metastasis in a follow-up examination. Interestingly this patient constitutes an outlier in terms of *DKK1* expression showing a down-regulation in the regional lymph nodes.

The results described in this section show that these metastasis-free pN1 lymph nodes exhibit gene regulations clearly different from those in pN0 lymph nodes. Furthermore they indicate that DKK1 might be significantly associated with the underlying mechanisms.

### The functional patterns in pN0 and pN1 samples are distinct but tightly connected

Based on these findings we asked whether the observed regulations indeed depict different molecular mechanisms – or merely different representatives of the same mechanisms. We therefore performed gene set enrichment and network analysis.

Because sample size used in our work is rather small the p-value distribution is affected in the T-test. Taking this into account and to allow for incorporation of more subtle changes, which nevertheless might add to the big picture, we decided to use a less stringent p-value of ±0.1 to identify regulated genes to be included in the analysis.

**Figure 3:**
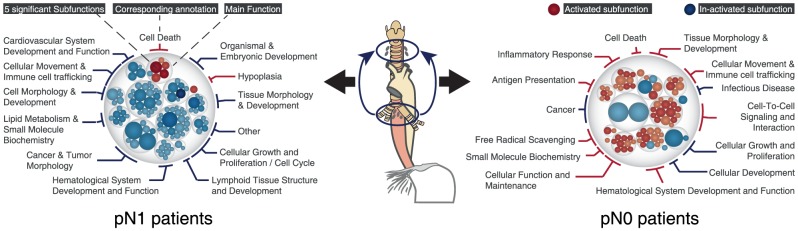
Functional gene set enrichment. Displayed are biological and molecular functions and sub-functions associated with the genes regulated in regional lymph nodes in comparison to the distant lymph nodes (reference) of pN1 or pN0 patients. The single transparent circles depict the main functions, the colored (red: activated, blue: inactivated) circles within depict the significantly enriched sub-functions (e.g. within pN1 cell death circle: ‘apoptosis of leukemia cell lines’ – red, or ‘cell survival’ – blue). Blue sub-functions have a strong prediction to be inactivated, red sub-functions to be activated. The deeper the color the more reliable (z-score) is the activation state's prediction. The size of the circles increases with the significance (p-value) of the gene list's association with the sub-functions. The analysis was performed with Ingenuity Pathway Analysis software. It can be clearly seen that metastasis-free regional lymph nodes of pN1 patients exhibit a completely different regulation-pattern that those of pN0 patients.

Our analysis revealed strikingly distinct profiles (Fig. 3) characterizing the tumor-adjacent lymph nodes of the pN1 and pN0 patients. The results showed a clear activation of immune response mechanisms in the pN0 group. The regulated genes were associated with enhanced inflammatory response, antigen presentation, reduced cellular growth, immune cell tracking and cell-to-cell signaling, while proliferation and cancer-associated mechanisms were reduced.

In contrast regional lymph nodes of pN1 patients exhibited a reduction of immune response associated mechanisms (Fig. 3). In addition, reduced proliferation as well as reduced tissue structure, morphology and development was observed on the one hand – and enhanced apoptosis and hypoplasia on the other. Time wise it should be expected that these lymph nodes would be closer to the point of metastatic invasion so it was surprising that the immune response pattern was down-regulated.

**Figure 4:**
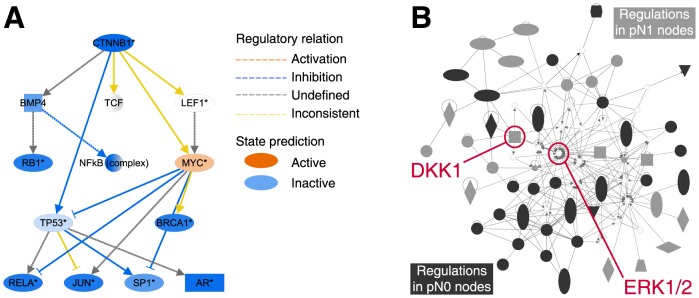
Network analysis. The network analysis was performed using Ingenuity Pathway Analysis software. **a**) Mechanistic network of β-catenin downstream regulators displaying predicted activation state and the regulatory relation (activation or inhibition) between the single regulators. β-catenin and a number of downstream regulators are predicted to be inactivated. **b)** The analysis shows a tight connection between the genes regulated in the pN1 samples (light grey nodes) and those regulated in the pN0 samples (dark grey nodes) linked by ERK1/2 and DKK1 embedded within the network.

Furthermore the analysis predicted an inactivation of β-catenin and downstream regulators (Fig. 4a). Among the major β-catenin targets, *VEGFA*, *CCND1*, *MSX1*, *IGFBP5 and DKK1* exhibited a decreased expression on the microarray. Though only based on statistical probabilities, the prediction is notable because active β-catenin is required for *DKK1* expression.[39] The potential inactivity of β-catenin therefore might correlate with the observed down-regulation of *DKK1*.

To further investigate how related the underlying molecular pathways are, we included the gene expression regulations observed in pN1 samples (109 genes) and in pN0 samples (128 genes) in one common list and performed network analysis. Interestingly, despite the clear difference in molecular mechanism profiles between pN1 and pN0 samples, the analysis revealed that several of the involved genes (28/128 pN0 genes; 19/109 pN1 genes) seemed to be tightly connected (Fig. 4b) across both groups – with DKK1 embedded within the network.

Taken together these observations suggest that metastasis-free regional lymph nodes of pN1 patients are already subject to molecular processes different to those of pN0 patients – though metastasis has not yet taken place in these nodes. In addition an involvement of *DKK1* regulation via the WNT/β-catenin pathway seems more plausible. This being said, the molecular networks underlying these different patterns might be connected. In ESC patients lymph node metastasis is only a question of time unless a timely tumor resection is performed. Therefore it seems plausible that the observed transcriptional patterns do not correlate with two totally different mechanisms but rather depict two time points of a progressing process.

### DKK1 can be observed in distinct substructures of the lymph node and in vessel endothelial cells

**Figure 5:**
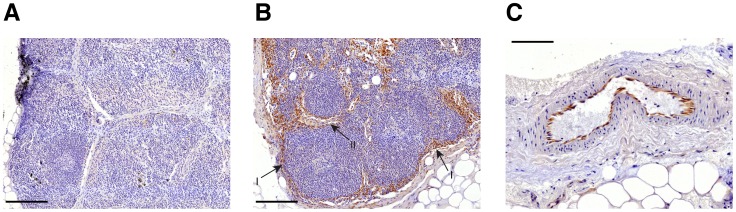
Representative immunohistochemical staining images of the investigated lymph nodes. Lymph nodes were stained using a polyclonal antibody against DKK1 (1∶100, abcam, #ab61034) and counterstained with hematoxylin. **a)** Representative DKK1 negative lymph node. Scale bar: 200 µm. **b)** Representative DKK1 positive lymph node. The subcapsular sinus is marked with (I) and the intermediary sinus and trabeculae with (II). Scale bar: 200 µm. **c)** DKK1 positive vascular endothelial cells. Scale bar: 100 µm.

Because of the significance of *DKK1* in our results and because DKK1 has already been shown to be prognostic for the outcome of a number of malignant tumor entities[30]-[38] we focused on this molecule in immunohistochemical validation. We stained paraffin sections of the investigated lymph nodes using a polyclonal antibody against DKK1 and counterstained the sections with hematoxylin. The immunohistochemical staining revealed a tendency of DKK1 to be less expressed in regional pN1 patients' lymph nodes (Fig. 5a). In DKK1 positive lymph nodes its expression could be clearly observed in the subcapsular sinus and in some cases extending into the intermediary sinus and trabeculae (Fig. 5b). Furthermore in a couple of samples DKK1 expression could be observed in vessel endothelial cells (Fig. 5c).

## Discussion

While several cancer incidence rates dropped in the United states and other Western countries over the last decades the incidence rate of esophageal adenocarcinoma has increased.[40] One parameters indicating disease progression is tumor expansion that currently forms the basis for therapy selection. Still, the molecular mechanisms of lymphogenic metastasis are mostly unknown. Several studies investigated the molecular profiles of esophageal cancer (ESC),[1]-[4] patient serum[37] and/or the invaded lymph nodes.[5] But to the authors best knowledge only few published studies investigated the pre-metastatic niche in lymph nodes of other cancers[13], [41], [42] in mice and none has investigated the metastatic niche for ESC prior to invasion in patients.

Our scope was to investigate this pre-metastatic niche in patients suffering from ESC and to determine the potential factors involved in early metastatic spread of tumor cells to lymph nodes. We hypothesized that the regional lymph nodes (adjacent to the tumor) might be more prone to be invaded first than the far lymph nodes. In consequence we used microarray technology to analyze these regional lymph nodes of ESC patients whom either suffered (pN1) or did not suffer (pN0) from lymphogenic metastasis at the time point of surgical intervention.

As there is evidence that squamous cell carcinoma (SCC) and adenocarcinoma (AC) of the esophagus might differ in their tumor progression one could imagine that these differences might have an impact on our observations. While SCCs are mainly observed in the proximal esophagus and are ascribed to smoking or alcohol abuse, ACs usually occur in the distal esophagus and are frequently preceded by Barrett's disease or gastroesophageal reflux disease. There are hints, that the tumor type might affect the location of lymph node metastasis.[43] However we assume that this is attributable to tumor location, not to histotype-specific molecular metastasis-pathways. In this study we assumed that both tumor histotypes might share some common molecular mechanisms within their metastatic cascade. To take this effect into account and to prevent a bias we distributed squamous cell carcinoma and adenocarcinoma over the analyzed groups (pN0 and pN1), see Table S1.

The sample size in our work (pN1 n = 9; pN0 n = 5) is small and the authors are aware that these numbers are below the power necessary for solid prognostic studies. This sample size goes back to the stringent quality selection process that the samples underwent before being included in our study. Only patients that did not undergo neoadjuvant radio-chemotherapy were included in the study. The lymph node segments dedicated for expression analysis were immediately dissected upon extraction in the operating room and directly transported in liquid nitrogen to the laboratory for RNA extraction. The other part of the lymph nodes was embedded in paraffin for diagnosis. And finally and foremost only metastasis- and micro-metastasis-free lymph nodes were included in the study, even for pN1 patients. The power of the work presented here is therefore the quality assurance process and the novel experimental design (see Fig. 1).

The results of this study suggest that regional lymph nodes of ESC patients might undergo alterations prior to metastatic invasion. This assumption is derived from the observation that lymph nodes of pN0 and pN1 patients exhibit clearly distinct transcriptional patterns (Fig. 3). While the transcriptional regulations in pN0 patients' lymph nodes clearly indicate an enhanced immune response those regulations observed in pN1 patients indicate a reduced immune response, reduced proliferation, reduced cancer associated mechanisms and enhanced apoptosis.

We assume that the enhanced immune response in the pN0 lymph nodes might result from a lymph node's exposure to circulating tumor cells or signaling processes of the primary tumor. An effective immune response might prevent these lymph nodes from metastatic invasion. The reduction of the immune response pattern in the pN1 lymph nodes might indicate a loss of this protective shield. This loss might provide circulating tumor cells an enhanced chance of survival. The observed reduction of proliferation and cancer & tumor morphology patterns as well as the enhanced apoptosis is primarily surprising and intriguing in terms of carcinogenesis. However, it has to be considered that at this point the investigated tissue constitutes the metastatic niche – not the metastasis itself or invaded lymph nodes. Increased apoptosis and reduced proliferation would suggest a morphological remodeling process in the lymph nodes that could facilitate invasion.

Approximately all ESC patients tend to develop metastasis if not treated in time. Therefore, we assume that these two different patterns do not reflect completely different molecular pathways but rather two different stages of a progressing disease. We assume that key molecules might mark the transition from one state to the next and that DKK1 might be one of these molecules.

An up-regulation of DKK1 has already been described in the context of diverse malignant tumors such as breast cancer, lung cancer, myeloma or even esophageal cancer.[30]-[38] In these studies DKK1 was investigated in context of the tumor itself or as molecule potentially secreted by the tumor into patients' serum. In breast cancer DKK1 is assumed to prepare the metastatic niche in the bone by deregulating the equilibrium between osteoclasts and osteoblasts.[44] These mechanisms however cannot be transferred to lymph nodes, especially because native DKK1 is down-regulated here.

Notably our immunohistochemical staining (Fig. 5) showed that before down-regulation, DKK1 could be observed not only within the subcapsular sinus and in parts within the intermediary sinus and trabeculae but also in vessel endothelial cells. Two recent studies established a link between WNT-signaling, extracellular matrix rigidity, barrier properties and invasion. Barbolina et al.[45] showed that matrix mechanical properties might regulate *DKK1* expression in epithelial ovarian cancer cells in vitro and that siRNA silencing of *DKK1* expression significantly enhanced invasion. Reis et al.[46] showed that Wnt-pathway inactivation in glioma via Dkk1 resulted in a higher vascular density and disrupted barrier function. They assumed that an active WNT signaling might be necessary for normalized and quiescent tumor vessels with increased barrier properties via PDGF-B expression induction.

The potential involvement of DKK1 and the WNT-pathway is further indicated by deregulation of associated genes (*MSX1, HOXA10, DKK1, MET* and *CCND1*) and by prediction of a β-catenin inactivation (Fig. 4a). MSX1 induces the WNT-pathway antagonist gene *DKK1*.[47] *DKK1* also exhibits binding sites for HOXA10.[48] Because DKK1 can inhibit the WNT/β-catenin pathway[49], [50] whereas its own expression is induced by the β-catenin/TCF complex[39], DKK1 imposes a negative force-feedback loop on itself[51] and can suppress expression of β-catenin target molecules such as *CCND1*. In breast cancer and colon cancer cells it has been found that inhibition of β-catenin might enhance invasive potential via upregulation of uPA/uPAR at mRNA and protein level[52]. Increased DKK1 serum concentrations have been observed in patients suffering from ESC[37], and high expression levels have been shown to be a prognostic biomarker for esophageal carcinomas[53]. In a very speculative outline tumor secreted DKK1 might potentially inhibit the WNT pathway in the regional lymph nodes thus inactivating β-catenin and down-regulating the lymph node native *DKK1* gene. In this case the tumor secreted DKK1 would probably not be observed in the immunohistochemical staining because it is bound to the receptors and might not be accessible by the antibody. That however would imply that the lymph node native DKK1 does not inhibit the WNT pathway.

A number of existing publications underscore that DKK1 might be a highly interesting diagnostic biomarker and therapeutic target. On diagnostic level DKK1 is reported as good serologic and prognostic biomarker for patients suffering from lung and esophageal carcinoma [37], as well as for patients with cervical cancer.[54] On therapeutic level a blockade of DKK1 using neutralizing antibodies (BHQ880, Novartis) was reported in a murine mouse model of multiple myeloma.[55] Furthermore DKK1 has been suggested as therapeutic target in patients suffering from breast cancer [56] or hepatocellular carcinoma [57], [58] and a patent claim [59] on “*using antibodies and antibody fragments capable of treating or preventing cancers associated with the over-expression and/or up-regulation of DKK1*“ is public since 2009.

In conclusion and to the authors' best knowledge, this is the first study to analyze the hypothesis of a tumor-adjacent lymphatic metastatic niche in esophageal carcinoma patients. Our findings strongly support the work published by Hirakawa et al.[13] and suggest that previous insights gained in mice also apply to patients. The results lead us to assume that preparation of a lymph metastatic niche takes place ahead of invasion and that this process progresses over more than one step. After initial immune response activation an advanced pre-invasive phenotype associated with WNT signaling deregulation, especially DKK1, might mark the lymph nodes' state on the verge of invasion.

The fact that metastasis-free lymph nodes already show alterations could change the view on the so-called local disease. On the long term it might have direct clinical impact and relevance for disease diagnosis and therapy selection. Our insights in future might a) help improving classification by better defining the intermediate stage between metastasis-free and invaded lymph nodes and b) help better identify patients with an increased metastatic risk that might need closer post-surgical observation or treatment, and c) help find therapies, which could reduce the risk of the “upcoming” metastatic invasion by restoring the lymph nodes' protective shield and finally d) raise awareness that targeted treatment of the metastatic niche alongside targeting the tumor cells might improve treatment outcome – a concept that potentially might be called “Assistance to Self-assistance”. The current paradigm in cancer treatment is targeting the tumor or metastatic cells. If a barrier is breached, however, or the environmental conditions in the niche are fit for metastatic invasion, then assisting classic targeted therapy by as well targeting these alterations within the metastatic niche might increase therapy effectiveness. Understanding the underlying mechanisms might help find solutions for such a niche-targeted therapy.

Until then our results should be validated in a bigger study setting and the precise role of DKK1 within lymph nodes remains to be investigated.

## Supporting Information

Table S1 **Detailed sample information.**
(XLSX)

Table S2 **Complete microarray differential regulation statistics.**
(XLSX)

Table S3 **Detailed PCR analysis results.**
(XLSM)
